# A systematic review on digital literacy

**DOI:** 10.1186/s40561-022-00204-y

**Published:** 2022-06-08

**Authors:** Hasan Tinmaz, Yoo-Taek Lee, Mina Fanea-Ivanovici, Hasnan Baber

**Affiliations:** 1 AI & Big Data Department, Endicott College of International Studies, Woosong University, Daejeon, South Korea; 2 Endicott College of International Studies, Woosong University, Daejeon, South Korea; 3 Department of Economics and Economic Policies, Bucharest University of Economic Studies, Bucharest, Romania; 4 Abu Dhabi School of Management, Abu Dhabi, United Arab Emirates

**Keywords:** Digital literacy, Digital competencies, Digital skills, Digital thinking, Systematic review, Qualitative research

## Abstract

The purpose of this study is to discover the main themes and categories of the research studies regarding digital literacy. To serve this purpose, the databases of WoS/Clarivate Analytics, Proquest Central, Emerald Management Journals, Jstor Business College Collections and Scopus/Elsevier were searched with four keyword-combinations and final forty-three articles were included in the dataset. The researchers applied a systematic literature review method to the dataset. The preliminary findings demonstrated that there is a growing prevalence of digital literacy articles starting from the year 2013. The dominant research methodology of the reviewed articles is qualitative. The four major themes revealed from the qualitative content analysis are: digital literacy, digital competencies, digital skills and digital thinking. Under each theme, the categories and their frequencies are analysed. Recommendations for further research and for real life implementations are generated.

## Introduction

The extant literature on digital literacy, skills and competencies is rich in definitions and classifications, but there is still no consensus on the larger themes and subsumed themes categories. (Heitin, 2016). To exemplify, existing inventories of Internet skills suffer from ‘incompleteness and over-simplification, conceptual ambiguity’ (van Deursen et al., 2015), and Internet skills are only a part of digital skills. While there is already a plethora of research in this field, this research paper hereby aims to provide a general framework of digital areas and themes that can best describe digital (cap)abilities in the novel context of Industry 4.0 and the accelerated pandemic-triggered digitalisation. The areas and themes can represent the starting point for drafting a contemporary digital literacy framework.

Sousa and Rocha (2019) explained that there is a stake of digital skills for disruptive digital business, and they connect it to the latest developments, such as the Internet of Things (IoT), cloud technology, big data, artificial intelligence, and robotics. The topic is even more important given the large disparities in digital literacy across regions (Tinmaz et al., 2022). More precisely, digital inequalities encompass skills, along with access, usage and self-perceptions. These inequalities need to be addressed, as they are credited with a ‘potential to shape life chances in multiple ways’ (Robinson et al., 2015), e.g., academic performance, labour market competitiveness, health, civic and political participation. Steps have been successfully taken to address physical access gaps, but skills gaps are still looming (Van Deursen & Van Dijk, 2010a). Moreover, digital inequalities have grown larger due to the COVID-19 pandemic, and they influenced the very state of health of the most vulnerable categories of population or their employability in a time when digital skills are required (Baber et al., 2022; Beaunoyer, Dupéré & Guitton, 2020).

The systematic review the researchers propose is a useful updated instrument of classification and inventory for digital literacy. Considering the latest developments in the economy and in line with current digitalisation needs, digitally literate population may assist policymakers in various fields, e.g., education, administration, healthcare system, and managers of companies and other concerned organisations that need to stay competitive and to employ competitive workforce. Therefore, it is indispensably vital to comprehend the big picture of digital literacy related research.

## Literature review

Since the advent of Digital Literacy, scholars have been concerned with identifying and classifying the various (cap)abilities related to its operation. Using the most cited academic papers in this stream of research, several classifications of digital-related literacies, competencies, and skills emerged.

### Digital literacies

Digital literacy, which is one of the challenges of integration of technology in academic courses (Blau, Shamir-Inbal & Avdiel, 2020), has been defined in the current literature as the competencies and skills required for navigating a fragmented and complex information ecosystem (Eshet, 2004). A ‘Digital Literacy Framework’ was designed by Eshet-Alkalai (2012), comprising six categories: (a) photo-visual thinking (understanding and using visual information); (b) real-time thinking (simultaneously processing a variety of stimuli); (c) information thinking (evaluating and combining information from multiple digital sources); (d) branching thinking (navigating in non-linear hyper-media environments); (e) reproduction thinking (creating outcomes using technological tools by designing new content or remixing existing digital content); (f) social-emotional thinking (understanding and applying cyberspace rules). According to Heitin (2016), digital literacy groups the following clusters: (a) finding and consuming digital content; (b) creating digital content; (c) communicating or sharing digital content. Hence, the literature describes the digital literacy in many ways by associating a set of various technical and non-technical elements.

### Digital competencies

The Digital Competence Framework for Citizens (DigComp 2.1.), the most recent framework proposed by the European Union, which is currently under review and undergoing an updating process, contains five competency areas: (a) information and data literacy, (b) communication and collaboration, (c) digital content creation, (d) safety, and (e) problem solving (Carretero, Vuorikari & Punie, 2017). Digital competency had previously been described in a technical fashion by Ferrari (2012) as a set comprising information skills, communication skills, content creation skills, safety skills, and problem-solving skills, which later outlined the areas of competence in DigComp 2.1, too.

### Digital skills

Ng (2012) pointed out the following three categories of digital skills: (a) technological (using technological tools); (b) cognitive (thinking critically when managing information); (c) social (communicating and socialising). A set of Internet skill was suggested by Van Deursen and Van Dijk (2009, 2010b), which contains: (a) operational skills (basic skills in using internet technology), (b) formal Internet skills (navigation and orientation skills); (c) information Internet skills (fulfilling information needs), and (d) strategic Internet skills (using the internet to reach goals). In 2014, the same authors added communication and content creation skills to the initial framework (van Dijk & van Deursen). Similarly, Helsper and Eynon (2013) put forward a set of four digital skills: technical, social, critical, and creative skills. Furthermore, van Deursen et al. (2015) built a set of items and factors to measure Internet skills: operational, information navigation, social, creative, mobile. More recent literature (vaan Laar et al., 2017) divides digital skills into seven core categories: technical, information management, communication, collaboration, creativity, critical thinking, and problem solving.

It is worth mentioning that the various methodologies used to classify digital literacy are overlapping or non-exhaustive, which confirms the conceptual ambiguity mentioned by van Deursen et al. (2015).

### Digital thinking

Thinking skills (along with digital skills) have been acknowledged to be a significant element of digital literacy in the educational process context (Ferrari, 2012). In fact, critical thinking, creativity, and innovation are at the very core of DigComp. Information and Communication Technology as a support for thinking is a learning objective in any school curriculum. In the same vein, analytical thinking and interdisciplinary thinking, which help solve problems, are yet other concerns of educators in the Industry 4.0 (Ozkan-Ozen & Kazancoglu, 2021).

However, we have recently witnessed a shift of focus from learning how to use information and communication technologies to using it while staying safe in the cyber-environment and being aware of alternative facts. Digital thinking would encompass identifying fake news, misinformation, and echo chambers (Sulzer, 2018). Not least important, concern about cybersecurity has grown especially in times of political, social or economic turmoil, such as the elections or the Covid-19 crisis (Sulzer, 2018; Puig, Blanco-Anaya & Perez-Maceira, 2021).

Ultimately, this systematic review paper focuses on the following major research questions as follows:

- Research question 1: What is the yearly distribution of digital literacy related papers?
- Research question 2: What are the research methods for digital literacy related papers?
- Research question 3: What are the main themes in digital literacy related papers?
- Research question 4: What are the concentrated categories (under revealed main themes) in digital literacy related papers?

## Method

This study employed the systematic review method where the authors scrutinized the existing literature around the major research question of digital literacy. As Uman (2011) pointed, in systematic literature review, the findings of the earlier research are examined for the identification of consistent and repetitive themes. The systematic review method differs from literature review with its well managed and highly organized qualitative scrutiny processes where researchers tend to cover less materials from fewer number of databases to write their literature review (Kowalczyk & Truluck, 2013; Robinson & Lowe, 2015).

### Data collection

To address major research objectives, the following five important databases are selected due to their digital literacy focused research dominance: 1. WoS/Clarivate Analytics, 2. Proquest Central; 3. Emerald Management Journals; 4. Jstor Business College Collections; 5. Scopus/Elsevier.

The search was made in the second half of June 2021, in abstract and key words written in English language. We only kept research articles and book chapters (herein referred to as papers). Our purpose was to identify a set of digital literacy areas, or an inventory of such areas and topics. To serve that purpose, systematic review was utilized with the following synonym key words for the search: ‘digital literacy’, ‘digital skills’, ‘digital competence’ and ‘digital fluency’, to find the mainstream literature dealing with the topic. These key words were unfolded as a result of the consultation with the subject matter experts (two board members from Korean Digital Literacy Association and two professors from technology studies department). Below are the four key word combinations used in the search: “Digital literacy AND systematic review”, “Digital skills AND systematic review”, “Digital competence AND systematic review”, and “Digital fluency AND systematic review”.

A sequential systematic search was made in the five databases mentioned above. Thus, from one database to another, duplicate papers were manually excluded in a cascade manner to extract only unique results and to make the research smoother to conduct. At this stage, we kept 47 papers. Further exclusion criteria were applied. Thus, only full-text items written in English were selected, and in doing so, three papers were excluded (no full text available), and one other paper was excluded because it was not written in English, but in Spanish. Therefore, we investigated a total number of 43 papers, as shown in Table 1. “Appendix A” shows the list of these papers with full references.

**Table 1:** Number of papers identified sequentially after applying all inclusion and exclusion criteria

Database	Keyword combinations				Total number of papers
	Digital literacy AND systematic review	Digital skills AND systematic review	Digital competence AND systematic review	Digital fluency AND systematic review	
1. WoS/Clarivate Analytics	4 papers	3 papers	5 papers	–	12 papers
2. Proquest Central	7 papers	4 papers	–	1 paper	12 papers
3.Emerald Management Jour	3 papers	1 paper	1 paper	-	5 papers
4. Jstor Business College Collection	9 papers	–	–	1 paper	10 papers
5. Scopus, Elsevier	4 papers	–	–	–	4 papers
Total per keyword combination	27 papers	8 papers	6 papers	2 papers	43 papers

### Data analysis

The 43 papers selected after the application of the inclusion and exclusion criteria, respectively, were reviewed the materials independently by two researchers who were from two different countries. The researchers identified all topics pertaining to digital literacy, as they appeared in the papers. Next, a third researcher independently analysed these findings by excluded duplicates A qualitative content analysis was manually performed by calculating the frequency of major themes in all papers, where the raw data was compared and contrasted (Fraenkel et al., 2012). All three reviewers independently list the words and how the context in which they appeared and then the three reviewers collectively decided for how it should be categorized. Lastly, it is vital to remind that literature review of this article was written after the identification of the themes appeared as a result of our qualitative analyses. Therefore, the authors decided to shape the literature review structure based on the themes.

## Results

**Fig. 1:**
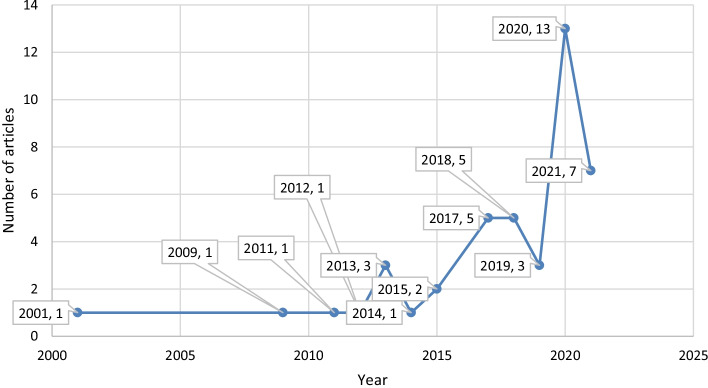
Yearly distribution of digital literacy related papers

As an answer to the first research question (the yearly distribution of digital literacy related papers), Fig. 1 demonstrates the yearly distribution of digital literacy related papers. It is seen that there is an increasing trend about the digital literacy papers.

**Fig. 2:**
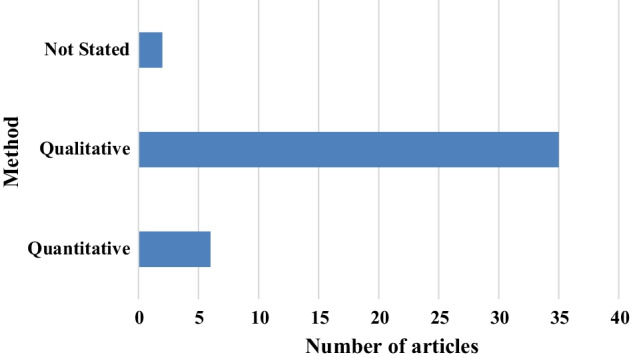
Research methods used in the reviewed articles

Research question number two (The research methods for digital literacy related papers) concentrates on what research methods are employed for these digital literacy related papers. As Fig. 2 shows, most of the papers were using the qualitative method. Not stated refers to book chapters.

When forty-three articles were analysed for the main themes as in research question number three (The main themes in digital literacy related papers), the overall findings were categorized around four major themes: (i) literacies, (ii) competencies, (iii) skills, and (iv) thinking. Under every major theme, the categories were listed and explained as in research question number four (The concentrated categories (under revealed main themes) in digital literacy related papers).

The authors utilized an overt categorization for the depiction of these major themes. For example, when the ‘creativity’ was labelled as a skill, the authors also categorized it under the ‘skills’ theme. Similarly, when ‘creativity’ was mentioned as a competency, the authors listed it under the ‘competencies’ theme. Therefore, it is possible to recognize the same finding under different major themes.

### Major theme 1: literacies

Digital literacy being the major concern of this paper was observed to be blatantly mentioned in five papers out forty-three. One of these articles described digital literacy as the human proficiencies to live, learn and work in the current digital society. In addition to these five articles, two additional papers used the same term as ‘critical digital literacy’ by describing it as a person’s or a society’s accessibility and assessment level interaction with digital technologies to utilize and/or create information. Table 2 summarizes the major categories under ‘Literacies’ major theme.

**Table 2:** Categories (more than one occurrence) under 'literacies' major theme

Category	n	Category	n	Category	n
Digital literacy	5	Disciplinary literacy	4	Web literacy	2
Critical digital literacy	2	Data literacy	3	New literacy	2
Computer literacy	5	Technology literacy	3	Mobile literacy	2
Media literacy	5	Multiliteracy	3	Personal literacy	2
Cultural literacy	5	Internet literacy	2	Research literacy	2

Computer literacy, media literacy and cultural literacy were the second most common literacy (n = 5). One of the article branches computer literacy as tool (detailing with software and hardware uses) and resource (focusing on information processing capacity of a computer) literacies. Cultural literacy was emphasized as a vital element for functioning in an intercultural team on a digital project.

Disciplinary literacy (n = 4) was referring to utilizing different computer programs (n = 2) or technical gadgets (n = 2) with a specific emphasis on required cognitive, affective and psychomotor skills to be able to work in any digital context (n = 3), serving for the using (n = 2), creating and applying (n = 2) digital literacy in real life.

Data literacy, technology literacy and multiliteracy were the third frequent categories (n = 3). The ‘multiliteracy’ was referring to the innate nature of digital technologies, which have been infused into many aspects of human lives.

Last but not least, Internet literacy, mobile literacy, web literacy, new literacy, personal literacy and research literacy were discussed in forty-three article findings. Web literacy was focusing on being able to connect with people on the web (n = 2), discover the web content (especially the navigation on a hyper-textual platform), and learn web related skills through practical web experiences. Personal literacy was highlighting digital identity management. Research literacy was not only concentrating on conducting scientific research ability but also finding available scholarship online.

Twenty-four other categories are unfolded from the results sections of forty-three articles. Table 3 presents the list of these other literacies where the authors sorted the categories in an ascending alphabetical order without any other sorting criterion. Primarily, search, tagging, filtering and attention literacies were mainly underlining their roles in information processing. Furthermore, social-structural literacy was indicated as the recognition of the social circumstances and generation of information. Another information-related literacy was pointed as publishing literacy, which is the ability to disseminate information via different digital channels.

**Table 3:** Other mentioned categories (n = 1)

Advanced digital assessment literacy	Intermediate digital assessment literacy	Search literacy
Attention literacy	Library literacy	Social media literacy
Basic digital assessment literacy	Metaliteracy	Social-structural literacy
Conventional print literacy	Multimodal literacy	Tagging literacy
Critical literacy	Network literacy	Television literacy
Emerging technology literacy	News literacy	Transcultural digital literacy
Film literacy	Participatory literacy	Transliteracy
Filtering literacy	Publishing literacy	

While above listed personal literacy was referring to digital identity management, network literacy was explained as someone’s social networking ability to manage the digital relationship with other people. Additionally, participatory literacy was defined as the necessary abilities to join an online team working on online content production.

Emerging technology literacy was stipulated as an essential ability to recognize and appreciate the most recent and innovative technologies in along with smart choices related to these technologies. Additionally, the critical literacy was added as an ability to make smart judgements on the cost benefit analysis of these recent technologies.

Last of all, basic, intermediate, and advanced digital assessment literacies were specified for educational institutions that are planning to integrate various digital tools to conduct instructional assessments in their bodies.

### Major theme 2: competencies

The second major theme was revealed as competencies. The authors directly categorized the findings that are specified with the word of competency. Table 4 summarizes the entire category set for the competencies major theme.

**Table 4:** Categories under 'competencies' major theme

Category	n	Category	n
Digital competence	14	Cross-cultural competencies	1
Digital competence as a life skill	5	Digital teaching competence	1
Digital competence for work	3	Balancing digital usage	1
Economic engagement	3	Political engagement	1
Digital competence for leisure	2	Complex system modelling competencies	1
Digital communication	2	Simulation competencies	1
Intercultural competencies	2	Digital nativity	1

The most common category was the ‘digital competence’ (n = 14) where one of the articles points to that category as ‘generic digital competence’ referring to someone’s creativity for multimedia development (video editing was emphasized). Under this broad category, the following sub-categories were associated:

- Problem solving (n = 10)
- Safety (n = 7)
- Information processing (n = 5)
- Content creation (n = 5)
- Communication (n = 2)
- Digital rights (n = 1)
- Digital emotional intelligence (n = 1)
- Digital teamwork (n = 1)
- Big data utilization (n = 1)
- Artificial Intelligence utilization (n = 1)
- Virtual leadership (n = 1)
- Self-disruption (in along with the pace of digitalization) (n = 1)

Like ‘digital competency’, five additional articles especially coined the term as ‘digital competence as a life skill’. Deeper analysis demonstrated the following points: social competences (n = 4), communication in mother tongue (n = 3) and foreign language (n = 2), entrepreneurship (n = 3), civic competence (n = 2), fundamental science (n = 1), technology (n = 1) and mathematics (n = 1) competences, learning to learn (n = 1) and self-initiative (n = 1).

Moreover, competencies were linked to workplace digital competencies in three articles and highlighted as significant for employability (n = 3) and ‘economic engagement’ (n = 3). Digital competencies were also detailed for leisure (n = 2) and communication (n = 2). Furthermore, two articles pointed digital competencies as an inter-cultural competency and one as a cross-cultural competency. Lastly, the ‘digital nativity’ (n = 1) was clarified as someone’s innate competency of being able to feel contented and satisfied with digital technologies.

### Major theme 3: skills

The third major observed theme was ‘skills’, which was dominantly gathered around information literacy skills (n = 19) and information and communication technologies skills (n = 18). Table 5 demonstrates the categories with more than one occurrence.

**Table 5:** Categories under 'skills' major theme

Category	n	Category	n
Information literacy skills	19	Decision making skills	3
ICT skills	18	Social intelligence	3
Communication skills	9	Digital learning	2
Collaboration skills	9	Digital teaching	2
Digital content creation skills	4	Digital fluency	2
Ethics for digital environment	4	Digital awareness	2
Research skills	3	Creativity	2

Table 6 summarizes the sub-categories of the two most frequent categories of ‘skills’ major theme. The information literacy skills noticeably concentrate on the steps of information processing; evaluation (n = 6), utilization (n = 4), finding (n = 3), locating (n = 2) information. Moreover, the importance of trial/error process, being a lifelong learner, feeling a need for information and so forth were evidently listed under this sub-category. On the other hand, ICT skills were grouped around cognitive and affective domains. For instance, while technical skills in general and use of social media, coding, multimedia, chat or emailing in specific were reported in cognitive domain, attitude, intention, and belief towards ICT were mentioned as the elements of affective domain.

**Table 6:** Sub-categories under ‘information literacy’ and ‘ICT’ skills

Sub-category for information literacy skills	n	Sub-category for ICT skills	n
Evaluating information	6	Technical skills	4
Using obtained information	4	Attitude towards ICT	4
Legal use of information	3	Use of social media	3
Finding information	3	Intention to use ICT	2
Locating information	2	Beliefs about the use of ICT	1
Feeling the need for information	1	General knowledge of ICT	1
Documenting information	1	Use of chat	1
Life-long learning	1	Use of email	1
Trial and error	1	Digital text skills	1
Dealing with the excessiveness of information	1	Use of multimedia technologies	1
		Coding	1

Communication skills (n = 9) were multi-dimensional for different societies, cultures, and globalized contexts, requiring linguistic skills. Collaboration skills (n = 9) are also recurrently cited with an explicit emphasis for virtual platforms.

‘Ethics for digital environment’ encapsulated ethical use of information (n = 4) and different technologies (n = 2), knowing digital laws (n = 2) and responsibilities (n = 2) in along with digital rights and obligations (n = 1), having digital awareness (n = 1), following digital etiquettes (n = 1), treating other people with respect (n = 1) including no cyber-bullying (n = 1) and no stealing or damaging other people (n = 1).

‘Digital fluency’ involved digital access (n = 2) by using different software and hardware (n = 2) in online platforms (n = 1) or communication tools (n = 1) or within programming environments (n = 1). Digital fluency also underlined following recent technological advancements (n = 1) and knowledge (n = 1) including digital health and wellness (n = 1) dimension.

‘Social intelligence’ related to understanding digital culture (n = 1), the concept of digital exclusion (n = 1) and digital divide (n = 3). ‘Research skills’ were detailed with searching academic information (n = 3) on databases such as Web of Science and Scopus (n = 2) and their citation, summarization, and quotation (n = 2).

‘Digital teaching’ was described as a skill (n = 2) in Table 4 whereas it was also labelled as a competence (n = 1) as shown in Table 3. Similarly, while learning to learn (n = 1) was coined under competencies in Table 3, digital learning (n = 2, Table 4) and life-long learning (n = 1, Table 5) were stated as learning related skills. Moreover, learning was used with the following three terms: learning readiness (n = 1), self-paced learning (n = 1) and learning flexibility (n = 1).

Table 7 shows other categories listed below the ‘skills’ major theme. The list covers not only the software such as GIS, text mining, mapping, or bibliometric analysis programs but also the conceptual skills such as the fourth industrial revolution and information management.

**Table 7:** Categories (one-time occurrence) under 'skills' major theme

Category	Category	Category
Digital connectivity skill	Culture transformation	Text mining
Digital systems skill	Readiness to Industry 4.0	GIS (geographic information system)
Re(design) skill	Internet of Things (IoT)	Bibliometric analysis
Digital readiness	Technology-human adaptation	Mapping
Digital commerce	Information management	

### Major theme 4: thinking

The last identified major theme was the different types of ‘thinking’. As Table 8 shows, ‘critical thinking’ was the most frequent thinking category (n = 4). Except computational thinking, the other categories were not detailed.

**Table 8:** Categories under ‘thinking’ major theme

Category	n	Category	n
Critical thinking	4	System thinking	1
Computational thinking	3	Interdisciplinary thinking	1
Analytical thinking	1	Purposeful thinking	1
Innovative thinking	1	Quick thinking	1

Computational thinking (n = 3) was associated with the general logic of how a computer works and sub-categorized into the following steps; construction of the problem (n = 3), abstraction (n = 1), disintegration of the problem (n = 2), data collection, (n = 2), data analysis (n = 2), algorithmic design (n = 2), parallelization & iteration (n = 1), automation (n = 1), generalization (n = 1), and evaluation (n = 2).

A transversal analysis of digital literacy categories reveals the following fields of digital literacy application:

- Technological advancement (IT, ICT, Industry 4.0, IoT, text mining, GIS, bibliometric analysis, mapping data, technology, AI, big data)
- Networking (Internet, web, connectivity, network, safety)
- Information (media, news, communication)
- Creative-cultural industries (culture, publishing, film, TV, leisure, content creation)
- Academia (research, documentation, library)
- Citizenship (participation, society, social intelligence, awareness, politics, rights, legal use, ethics)
- Education (life skills, problem solving, teaching, learning, education, lifelong learning)
- Professional life (work, teamwork, collaboration, economy, commerce, leadership, decision making)
- Personal level (critical thinking, evaluation, analytical thinking, innovative thinking)

## Discussion

This systematic review on digital literacy concentrated on forty-three articles from the databases of WoS/Clarivate Analytics, Proquest Central, Emerald Management Journals, Jstor Business College Collections and Scopus/Elsevier. The initial results revealed that there is an increasing trend on digital literacy focused academic papers. Research work in digital literacy is critical in a context of disruptive digital business, and more recently, the pandemic-triggered accelerated digitalisation (Beaunoyer, Dupéré & Guitton, 2020; Sousa & Rocha 2019). Moreover, most of these papers were employing qualitative research methods. The raw data of these articles were analysed qualitatively using systematic literature review to reveal major themes and categories. Four major themes that appeared are: digital literacy, digital competencies, digital skills and thinking.

Whereas the mainstream literature describes digital literacy as a set of photo-visual, real-time, information, branching, reproduction and social-emotional thinking (Eshet-Alkalai, 2012) or as a set of precise specific operations, i.e., finding, consuming, creating, communicating and sharing digital content (Heitin, 2016), this study reveals that digital literacy revolves around and is in connection with the concepts of computer literacy, media literacy, cultural literacy or disciplinary literacy. In other words, the present systematic review indicates that digital literacy is far broader than specific tasks, englobing the entire sphere of computer operation and media use in a cultural context.

The digital competence yardstick, DigComp (Carretero, Vuorikari & Punie, 2017) suggests that the main digital competencies cover information and data literacy, communication and collaboration, digital content creation, safety, and problem solving. Similarly, the findings of this research place digital competencies in relation to problem solving, safety, information processing, content creation and communication. Therefore, the findings of the systematic literature review are, to a large extent, in line with the existing framework used in the European Union.

The investigation of the main keywords associated with digital skills has revealed that information literacy, ICT, communication, collaboration, digital content creation, research and decision-making skill are the most representative. In a structured way, the existing literature groups these skills in technological, cognitive, and social (Ng, 2012) or, more extensively, into operational, formal, information Internet, strategic, communication and content creation (van Dijk & van Deursen, 2014). In time, the literature has become richer in frameworks, and prolific authors have improved their results. As such, more recent research (vaan Laar et al., 2017) use the following categories: technical, information management, communication, collaboration, creativity, critical thinking, and problem solving.

Whereas digital thinking was observed to be mostly related with critical thinking and computational thinking, DigComp connects it with critical thinking, creativity, and innovation, on the one hand, and researchers highlight fake news, misinformation, cybersecurity, and echo chambers as exponents of digital thinking, on the other hand (Sulzer, 2018; Puig, Blanco-Anaya & Perez-Maceira, 2021).

## Conclusion

This systematic review research study looks ahead to offer an initial step and guideline for the development of a more contemporary digital literacy framework including digital literacy major themes and factors. The researchers provide the following recommendations for both researchers and practitioners.

### Recommendations for prospective research

By considering the major qualitative research trend, it seems apparent that more quantitative research-oriented studies are needed. Although it requires more effort and time, mixed method studies will help understand digital literacy holistically.

As digital literacy is an umbrella term for many different technologies, specific case studies need be designed, such as digital literacy for artificial intelligence or digital literacy for drones’ usage.

Digital literacy affects different areas of human lives, such as education, business, health, governance, and so forth. Therefore, different case studies could be carried out for each of these unique dimensions of our lives. For instance, it is worth investigating the role of digital literacy on lifelong learning in particular, and on education in general, as well as the digital upskilling effects on the labour market flexibility.

Further experimental studies on digital literacy are necessary to realize how certain variables (for instance, age, gender, socioeconomic status, cognitive abilities, etc.) affect this concept overtly or covertly. Moreover, the digital divide issue needs to be analysed through the lens of its main determinants.

New bibliometric analysis method can be implemented on digital literacy documents to reveal more information on how these works are related or centred on what major topic. This visual approach will assist to realize the big picture within the digital literacy framework.

### Recommendations for practitioners

The digital literacy stakeholders, policymakers in education and managers in private organizations, need to be aware that there are many dimensions and variables regarding the implementation of digital literacy. In that case, stakeholders must comprehend their beneficiaries or the participants more deeply to increase the effect of digital literacy related activities. For example, critical thinking and problem-solving skills and abilities are mentioned to affect digital literacy. Hence, stakeholders have to initially understand whether the participants have enough entry level critical thinking and problem solving.

Development of digital literacy for different groups of people requires more energy, since each group might require a different set of skills, abilities, or competencies. Hence, different subject matter experts, such as technologists, instructional designers, content experts, should join the team.

It is indispensably vital to develop different digital frameworks for different technologies (basic or advanced) or different contexts (different levels of schooling or various industries).

These frameworks should be updated regularly as digital fields are evolving rapidly. Every year, committees should gather around to understand new technological trends and decide whether they should address the changes into their frameworks.

Understanding digital literacy in a thorough manner can enable decision makers to correctly implement and apply policies addressing the digital divide that is reflected onto various aspects of life, e.g., health, employment, education, especially in turbulent times such as the COVID-19 pandemic is.

Lastly, it is also essential to state the study limitations. This study is limited to the analysis of a certain number of papers, obtained from using the selected keywords and databases. Therefore, an extension can be made by adding other keywords and searching other databases.

## Data Availability

The authors present the articles used for the study in “Appendix A”.

## Appendix A

See Table

**Table 9:** List of papers (n = 43) included in the qualitative analysis—ordered alphabetically by title

#	Author and year	Title	Journal/Book
1	Sulzer, M. A. (2018)	(Re)conceptualizing digital literacies before and after the election of Trump	English Teaching: Practice and Critique
2	Gunduzalp, S. (2021)	21st Century Skills for Sustainable Education: Prediction Level of Teachers’ Information Literacy Skills on Their Digital Literacy Skills	Discourse and Communication for Sustainable Education
3	Palts, T., Pedaste, M. (2020)	A Model for Developing Computational Thinking Skills	Informatics in Education
4	Starkey, L. (2020)	A systematic review of research exploring teacher preparation for the digital age	Cambridge Journal of Education
5	Ozkan-Ozen, Y. D., Kazancoglu, Y. (2021)	Analysing workforce development challenges in the Industry 4.0	International Journal of Manpower
6	Barna, C., Epure, M. (2020)	Analyzing youth unemployment and digital literacy skills in romania in the context of the current digital transformation	Review of Applied Socio-Economic Research
7	Reis, D. A., Fleury, A. L., Carvalho, M. M. (2021)	Consolidating core entrepreneurial competences: toward a meta-competence framework	International Journal of Enterpreneurial Behavior & Researh
8	van Laar, E., van Deursen, J. A. M., van Dijk, J. A. G. M., de Haan, J. (2020)	Determinants of 21st-Century Skills and 21st-Century Digital Skills for Workers: A Systematic Literature Review	SAGE Open
9	Kim, M., Choi, D. (2018)	Development of Youth Digital Citizenship Scale and Implication for Educational Setting	Journal of Educational Technology & Society
10	Eyal, L. (2012)	Digital Assessment Literacy — the Core Role of the Teacher in a Digital Environment	Journal of Educational Technology & Society
11	Spante, M., Hashemi, S. S., Lundin, M., Algers, A. (2018)	Digital competence and digital literacy in higher education research: Systematic review of concept use	Cogent Education
12	Zhao, Y., Pinto Llorente, A. M., Cruz Sanchez Gomez, M. (2021)	Digital competence in higher education research: A systematic literature review	Computers & Education
13	Batanero, J. M. F., Montenegro Rueda, M., Cerero, J. F., Garcia Martinez, I. (2020)	Digital competences for teacher professional development. Systematic review	European Journal of Teacher Education
14	Murawski, M., Bick, M. (2017)	Digital competences of the workforce – a research topic?	Business Process Management Journal
15	Gibson, P. F., Smith, S. (2018)	Digital literacies: preparing pupils and students for their information journey in the twenty-first century	Information and Learning Science
16	Mcclurken, J., Boggs, J., Wadewitz, A., Geller, A. E., Beasley-Murray, J. (2013)	Digital Literacy and the Undergraduate Curriculum	Book: Hacking the Academy: New Approaches to Scholarship and Teaching from Digital Humanities. The University of Michigan Press
17	Radovanovic, D., Holst, C., Belur, S. B., Srivastava, R., Houngbonon, G. V., Le Quentrec, E., Miliza, J., Winkler, A. S., Noll, J. (2020)	Digital Literacy Key Performance Indicators for Sustainable Development	Social Inclusion
18	Soomro, M. A., Hizam-Hanafiah, M., Abdullah, N. L. (2020)	Digital readiness models: A systematic literature review	Compusoft, An international journal of advanced computer technology
19	Martinez-Bravo, M. C., Sadaba-Chalezquer, C., Serrano-Puche, J. (2020)	Fifty years of digital literacy studies: A meta-research for interdisciplinary and conceptual convergence	Profesional de la informacion
20	Kolle, S. R. (2017)	Global research on information literacy: a bibliometric analysis from 2005 to 2014	The Electronic Library
21	Dominguez Figaredo, D. (2017)	Heuristics and Web Skills Acquisition in Open Learning Environments	Educational Technology & Society
22	Bawden, D. (2001)	Information and digital literacies: a review of concepts	Journal of Documentation
23	Coklar, A. N., Yaman, N. D., Yurdakul, I. K. (2017)	Information literacy and digital nativity as determinants of online information search strategies	Computers in Human Behavior
24	Fosmire, M. (2014)	Information literacy and lifelong learning	Book: Integrating Information into the Engineering Design Process, Purdue University Press
25	Buschman, J. (2009)	Information Literacy, “New” Literacies, and Literacy	The Library Quarterly
26	Reis, C., Pessoa, T., Gallego-Arrufat, M. J. (2019)	Literacy and digital competence in Higher Education: A systematic review	Revista de Docencia Universitaria
27	Oh, S. S., Kim, K.-A., Kim, M., Oh, J., Chu, S. H., Choi, J. Y. (2021)	Measurement of Digital Literacy Among Older Adults: Systematic Review	Journal of Medical Internet Research
28	Santandreu Calonge, D., Shah, M. A., Riggs, K., Connor, M. (2019)	MOOCs and upskilling in Australia: A qualitative literature study	Cogent Education
29	Mahiri, J. (2011)	New literacies need new learning	Book: Digital Tools in Urban Schools. Mediating a Remix of Learning. The University of Michigan Press
30	Hicks, T., Hawley Turner, K. (2013)	No Longer a Luxury: Digital Literacy Can't Wait	English Journal
31	Khuraisah, M. N., Khalid, F., Husnin, H. (2020)	Preparing graduates with digital literacy skills toward fulfilling employability need in 4IR Era: A review	International Journal of Advanced Computer Science and Applications
32	da Silva, C. R. S., Carvalho Teixeira, T. M., Bentes Pinto, V. (2019)	Research methodology in information literacy: A systematic review	Digital Journal of Library and Information Science
33	Garcia-Perez, L., Garcia-Garnica, M., Olmedo-Moreno, E. M. (2021)	Skills for a Working Future: How to Bring about Professional Success from the Educational Setting	Education Sciences
34	Stordy, P. (2015)	Taxonomy of literacies	Journal of Documentation
35	Aesaert, K., Vanderlinde, R., Tondeur, J., van Braak, J. (2013)	The content of educational technology curricula: a cross-curricular state of the art	Educational Technology Research and Development
36	de Greef, M., Segers, M., Nijhuis, J., Lam, J. F., van Groenestijn, M., van Hoek, F., van Deursen, A. J. A. M., Bohnenn, E., Tubbing, M. (2015)	The development and validation of testing materials for literacy, numeracy and digital skills in a Dutch context	International Review of Education
37	Rodriguez-Garcia, A. M., Caceres Reche, M. P., Garcia, S. A. (2018)	The digital competence of the future teacher: bibliometric analysis of scientific productivity indexed in Scopus	International Journal of Educational Research and Innovation
38	Sanchez-Caballe, A., Gisbert-Cervera, M., Esteve-Mon, F. (2020)	The digital competence of university students: a systematic literature review	Aloma
39	Keshavarz, M. (2020)	The effect of distance education on information literacy case study: Iran	The Quarterly Review of Distance Education
40	van Laar, E., van Deursen, J. A. M., van Dijk, J. A. G. M., de Haan, J.(2017)	The relation between 21st-century skills and digital skills: A systematic literature review	Computers in Human Behavior
41	Esteban-Navarro, M. A., Garcia-Madurga, M. A., Morte-Nadal, T., Nogales-Bocio, A. I. (2020)	The Rural Digital Divide in the Face of the COVID-19 Pandemic in Europe—Recommendations from a Scoping Review	Informatics
42	Rifai, I., Setiadi, C. J., Renaldo, J. Andreani, W. (2021)	Toward society 5.0: Indonesia and Japan on the twenty-first century literacy skills	IOP Conf. Series: Earth and Environmental Science
43	Kozanoglu, D. C., Abedin, B. (2020)	Understanding the role of employees in digital transformation: conceptualization of digital literacy of employees as a multi-dimensional organizational affordance	Journal of Enterprise Information Management

9.

## References

[CR1] Baber, H., Fanea-Ivanovici, M., Lee, Y. T., & Tinmaz, H. (2022). A bibliometric analysis of digital literacy research and emerging themes pre-during COVID-19 pandemic. *Information and Learning Sciences*. 10.1108/ILS-10-2021-0090.

[CR2] Beaunoyer, E., Dupéré, S., & Guitton, M. J. (2020). COVID-19 and digital inequalities: Reciprocal impacts and mitigation strategies. *Computers in Human Behavior,**111*, 10642. 10.1016/j.chb.2020.106424 10.1016/j.chb.2020.106424 PMC7213963 32398890

[CR3] Blau, I., Shamir-Inbal, T., & Avdiel, O. (2020). How does the pedagogical design of a technology-enhanced collaborative academic course promote digital literacies, self-regulation, and perceived learning of students? *The Internet and Higher Education,**45*, 100722. 10.1016/j.iheduc.2019.100722

[CR4] Carretero, S., Vuorikari, R., & Punie, Y. (2017). *DigComp 2.1: The Digital Competence Framework for Citizens with eight proficiency levels and examples of use (No. JRC106281).* Joint Research Centre, https://publications.jrc.ec.europa.eu/repository/handle/JRC106281

[CR5] Eshet, Y. (2004). Digital literacy: A conceptual framework for survival skills in the digital era. *Journal of Educational Multimedia and Hypermedia*, *13*(1), 93–106, https://www.learntechlib.org/primary/p/4793/

[CR6] Eshet-Alkalai, Y. (2012). Thinking in the digital era: A revised model for digital literacy. *Issues in Informing Science and Information Technology,**9*(2), 267–276. 10.28945/1621

[CR7] Ferrari, A. (2012). *Digital competence in practice: An analysis of frameworks.* JCR IPTS, Sevilla. https://ifap.ru/library/book522.pdf

[CR8] Fraenkel, J. R., Wallen, N. E., & Hyun, H. H. (2012). *How to design and evaluate research in education* (8th ed.). Mc Graw Hill.

[CR9] Heitin, L. (2016). What is digital literacy? *Education Week,* https://www.edweek.org/teaching-learning/what-is-digital-literacy/2016/11

[CR10] Helsper, E. J., & Eynon, R. (2013). Distinct skill pathways to digital engagement. *European Journal of Communication,**28*(6), 696–713. 10.1177/0267323113499113

[CR11] Kowalczyk, N., & Truluck, C. (2013). Literature reviews and systematic reviews: What is the difference*?*. *Radiologic Technology,**85*(2), 219–222. 24255144

[CR12] Ng, W. (2012). Can we teach digital natives digital literacy? *Computers & Education,**59*(3), 1065–1078. 10.1016/j.compedu.2012.04.016

[CR13] Ozkan-Ozen, Y. D., & Kazancoglu, Y. (2021). Analysing workforce development challenges in the Industry 4.0. *International Journal of Manpower*. 10.1108/IJM-03-2021-0167

[CR14] Puig, B., Blanco-Anaya, P., & Perez-Maceira, J. J. (2021). “Fake News” or Real Science? Critical thinking to assess information on COVID-19. *Frontiers in Education,**6*, 646909. 10.3389/feduc.2021.646909

[CR15] Robinson, L., Cotten, S. R., Ono, H., Quan-Haase, A., Mesch, G., Chen, W., Schulz, J., Hale, T. M., & Stern, M. J. (2015). Digital inequalities and why they matter. *Information, Communication & Society,**18*(5), 569–582. 10.1080/1369118X.2015.1012532

[CR16] Robinson, P., & Lowe, J. (2015). Literature reviews vs systematic reviews. *Australian and New Zealand Journal of Public Health,**39*(2), 103. 10.1111/1753-6405.12393 25827181 10.1111/1753-6405.12393

[CR17] Sousa, M. J., & Rocha, A. (2019). Skills for disruptive digital business. *Journal of Business Research,**94*, 257–263. 10.1016/j.jbusres.2017.12.051

[CR18] Sulzer, A. (2018). (Re)conceptualizing digital literacies before and after the election of Trump. *English Teaching: Practice & Critique,**17*(2), 58–71. 10.1108/ETPC-06-2017-0098

[CR19] Tinmaz, H., Fanea-Ivanovici, M., & Baber, H. (2022). A snapshot of digital literacy. *Library Hi Tech News*, (ahead-of-print).

[CR20] Uman, L. S. (2011). Systematic reviews and meta-analyses. *Journal of the Canadian Academy of Child and Adolescent Psychiatry,**20*(1), 57–59. 21286370 PMC3024725

[CR21] Van Deursen, A. J. A. M., Helsper, E. J., & Eynon, R. (2015). Development and validation of the Internet Skills Scale (ISS). *Information, Communication & Society,**19*(6), 804–823. 10.1080/1369118X.2015.1078834

[CR22] Van Deursen, A. J. A. M., & van Dijk, J. A. G. M. (2009). Using the internet: Skills related problems in users’ online behaviour. *Interacting with Computers,**21*, 393–402. 10.1016/j.intcom.2009.06.005

[CR23] Van Deursen, A. J. A. M., & van Dijk, J. A. G. M. (2010a). Measuring internet skills. *International Journal of Human-Computer Interaction,**26*(10), 891–916. 10.1080/10447318.2010.496338

[CR24] Van Deursen, A. J. A. M., & van Dijk, J. A. G. M. (2010b). Internet skills and the digital divide. *New Media & Society,**13*(6), 893–911. 10.1177/1461444810386774

[CR25] van Dijk, J. A. G. M., & Van Deursen, A. J. A. M. (2014). *Digital skills, unlocking the information society*. Palgrave MacMillan.

[CR26] van Laar, E., van Deursen, A. J. A. M., van Dijk, J. A. G. M., & de Haan, J. (2017). The relation between 21st-century skills and digital skills: A systematic literature review. *Computer in Human Behavior,**72*, 577–588. 10.1016/j.chb.2017.03.010

